# Cancer Metabolism: A Modeling Perspective

**DOI:** 10.3389/fphys.2015.00382

**Published:** 2015-12-16

**Authors:** Pouyan Ghaffari, Adil Mardinoglu, Jens Nielsen

**Affiliations:** ^1^Department of Biology and Biological Engineering, Chalmers University of TechnologyGothenburg, Sweden; ^2^Science for Life Laboratory, KTH - Royal Institute of TechnologyStockholm, Sweden; ^3^Novo Nordisk Foundation Center for Biosustainability, Technical University of DenmarkHørsholm, Denmark

**Keywords:** cancer metabolism, metabolic modeling, systems biology, systems medicine, genome scale metabolic reconstruction, metabolic networks and pathways, tumor metabolism

## Abstract

Tumor cells alter their metabolism to maintain unregulated cellular proliferation and survival, but this transformation leaves them reliant on constant supply of nutrients and energy. In addition to the widely studied dysregulated glucose metabolism to fuel tumor cell growth, accumulating evidences suggest that utilization of amino acids and lipids contributes significantly to cancer cell metabolism. Also recent progresses in our understanding of carcinogenesis have revealed that cancer is a complex disease and cannot be understood through simple investigation of genetic mutations of cancerous cells. Cancer cells present in complex tumor tissues communicate with the surrounding microenvironment and develop traits which promote their growth, survival, and metastasis. Decoding the full scope and targeting dysregulated metabolic pathways that support neoplastic transformations and their preservation requires both the advancement of experimental technologies for more comprehensive measurement of omics as well as the advancement of robust computational methods for accurate analysis of the generated data. Here, we review cancer-associated reprogramming of metabolism and highlight the capability of genome-scale metabolic modeling approaches in perceiving a system-level perspective of cancer metabolism and in detecting novel selective drug targets.

## Introduction

The past decades has seen a dramatic expansion in investigations on mechanism of cancer related metabolic adaptations, and this has resulted in accumulated evidences suggesting considerable association between several pathways in human metabolism and malignant transformation (Vander Heiden et al., 2009; Cairns et al., 2011; Schulze and Harris, 2012). Recently, the state of “deregulated cellular metabolism” was added by Hanahan and Weinberg as one of the hallmarks of cancer (Hanahan and Weinberg, 2000), reflecting the overall consensus around the idea of altered cellular metabolism through neoplastic progression (Hanahan and Weinberg, 2011). Consistent with this approach, investigation of cancer-associated metabolic alterations attracted considerable effort and resulted in successful identification of several selective metabolic targets that started to enter clinical trials (Vander Heiden, 2011; Galluzzi et al., 2013). Activation of oncogenes and deactivation of tumor suppressor genes, have been linked to cancer-associated metabolic reprogramming (Levine and Puzio-Kuter, 2010). Also, accumulation of metabolites such as 2-hydroxyglutarate, fumarate, and succinate has been proposed to drive oncogenesis, due to disruption in enzymatic activity of isocitrate dehydrogenase (IDH), fumarate hydratase (FH), and succinate dehydrogenase (SDH), respectively (Isaacs et al., 2005; Selak et al., 2005; Dang et al., 2010). Additionally, some agents that conventionally have been used in cancer treatment–such as gemcitabine, 5-fluourouracil and methotrexate—are in fact metabolic enzyme inhibitors (Chabner and Roberts, 2005). In line with these findings, accumulated evidences from recent epidemiological studies supported influence of whole-body metabolism on tumor progression and drug response (Vander Heiden et al., 2009; Vander Heiden, 2011; Galluzzi et al., 2013).

High-throughput omics technologies experienced a dramatic breakthrough during the last decade enabling simultaneous measurement of interacting molecular components in context of complex cellular structure and characterization of transformed cellular processes at genome-scale. Accumulation of DNA, microRNA and protein expression measurements, along with more detailed metabolomics data, has revealed a broader view on cancer-associated metabolic shifts. The more advanced understanding of molecular and genetic events underlying neoplastic transformation acquired, the more complex portrait of tumor metabolism has emerged. Today, analyzing multi-layer data generated via high-throughput technologies and integrating them into a descriptive unified model is a nontrivial challenge. Computational methods, capable of processing and integrating multi-dimensional data, have been employed in investigation of metabolic states in health and disease, and in identification of new selective targets and biomarkers. Here, we will review current knowledge in cancer metabolism, underlining capabilities of genome-wide metabolic models in opening new therapeutic windows.

## Fuels for cancer cells

One of the best known features of most tumor cells is utilizing high amounts of glucose and metabolizing it differently from normal cells, converting pyruvate derived from glucose to lactate in the cytosol and secreting it rather than oxidizing pyruvate in mitochondria. Normal cells principally elevate conversion of glucose to lactate in hypoxic conditions, whereas this phenotype is common in transformed cancer cells even when oxygen is abundant, a phenotype first described by Otto Warburg more than fifty years ago and referred to as “The Warburg effect” or aerobic glycolysis (Warburg, 1956). Glycolysis can produce ATP faster but far less efficient than oxidative phosphorylation. This shift makes tumor cells to be dependent on high rate of glucose consumption to meet their biosynthesis, energy, and redox requirements (Semenza et al., 2001; Cairns et al., 2011). This metabolic phenotype of cancer cells, dramatic increase in glucose uptake, has been studied extensively and used clinically in cancer diagnosis to visualize tumors by 2-deoxy-2-[fluorine-18] fluoro-D-glucose positron emission tomography (^18^F-FDG PET; Som et al., 1980; Kelloff et al., 2005).

**Figure 1:**
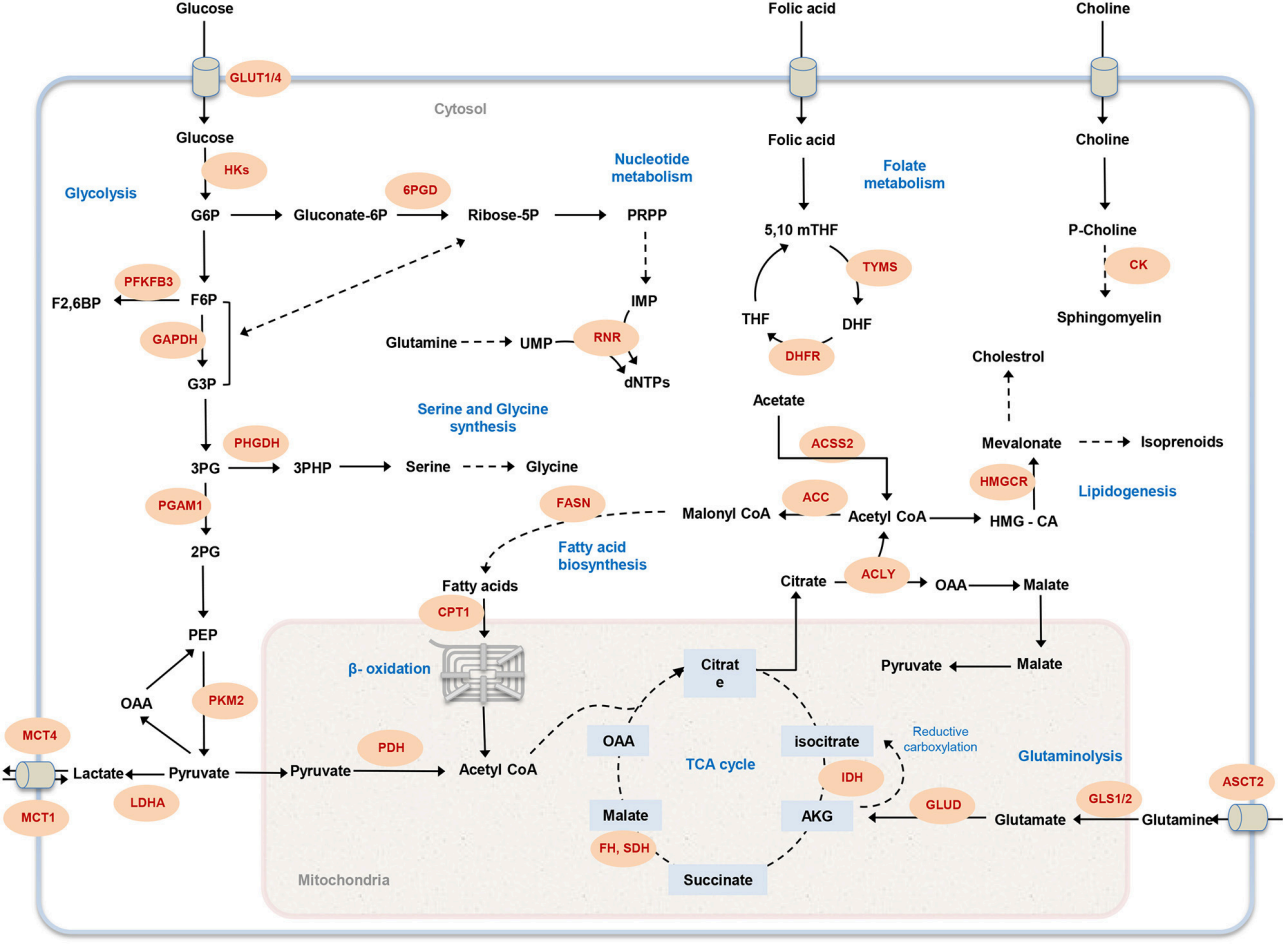
**Overview of cancer-associated metabolic pathways**. The main metabolic pathways that contributes to malignancy and offer potential drug targets are illustrated. Metabolic enzymes that have been associated with tumor initiation and growth are marked in red. GLUT1, glucose transporter; HK, hexokinase; 6PGD, 6-Phosphogluconate dehydrogenase; PFKFB3, 6-phosphofructo-2-kinase; GAPDH, Glyceraldehyde 3-phosphate dehydrogenase; PHGDH, phosphoglycerate dehydrogenase; PGAM1, Phosphoglycerate mutase 1; PKM, Pyruvate kinase; LDHA, lactate dehydrogenase A; MCT, Monocarboxylate transporter; PDH, Pyruvate dehydrogenase; CPT1, Carnitine palmitoyltransferase I; FASN, fatty acid synthase; RNR, ribonucleotide reductase; FH, fumarate hydratase; SDH, succinate dehydrogenase; IDH, isocitrate dehydrogenase; GLUD, glutamate dehydrogenase; GLS1, glutaminase 1; ASCT2, Amino-acid transporter 2; ACLY, ATP citrate lyase; ACC, acetyl-CoA carboxylase; ACSS2, Acetyl CoA synthetase2; DHFR, DHF reductase; TYMS, thymidylate synthase; HMGCR, HMG-CoA reductase; CK, choline kinase; G6P, glucose-6-phosphate; F6P, fructose-6-phosphate; PRPP, 5-phospho-alpha-D-ribose 1-diphosphate; IMP, inosine monophosphate; UMP, uridine monophosphate; dNTP, deoxynucleotide triphosphate; G3P, glyceraldehyde 3-phosphate; 3PG, 3-phosphoglycerate; 2PG, 2-phosphoglycerate; PEP, phosphoenolpyruvate; OAA, Oxaloacetate; AKG, α-ketoglutarate; HMG-CoA, 3-hydroxy-3-methyl-glutaryl coenzyme A; THF, tetrahydrofolate; 5,10 mTHF, 5,10-methylene tetrahydrofolate; DHF, dihydrofolate.

Elevated glycolytic flux promotes shunting of compounds into branched metabolic pathways to synthesis macromolecules needed for proliferation (Cairns et al., 2011). Downstream flux of glycolysis intermediates into oxidative and non-oxidative arms of the pentose phosphate pathway (PPP) produces ribose-5-phosphate and NADPH, two essential components for tumor cell growth. Ribose-5-phosphate is a precursor for nucleotide synthesis and NADPH is required to handle redox stress (Lunt and Vander Heiden, 2011; Dang, 2012). Another branched pathway from glycolysis is serine biosynthesis, which is important for nucleotide, amino acid, and lipid biosynthesis (Lunt and Vander Heiden, 2011). Sustained proliferation of some melanoma and breast cancer cells has been associated to amplification of phosphoglycerate dehydrogenase (PHGDH), the enzyme catalyzing the first step of serine biosynthesis (Locasale et al., 2011; Possemato et al., 2011). Furthermore, the metastasis of breast cancer cell has been associated with the up-regulation of the serine biosynthesis pathway (Possemato et al., 2011; Figure 1).

Glutamine plays a key role in sustaining rapid cell proliferation (Jain et al., 2012). Glutamine is the most abundant amino acid in culture media (Eagle et al., 1956) and plasma (Stein and Moore, 1954) and seems to be in excess relative to required amounts for protein and nucleotide synthesis (DeBerardinis et al., 2007). Tumor cells experiencing aerobic glycolysis need glutamine carbon to replenish TCA cycle intermediates and sustain enhanced biosynthetic metabolism to support cell proliferation (DeBerardinis et al., 2007; Lunt and Vander Heiden, 2011; Mullen et al., 2012). Glutamine is also a primary source of nitrogen for the cell (DeBerardinis et al., 2007). Under hypoxic conditions, glutamine can undergo reductive deamination to generate alpha-ketoglutrate and consequently oxaloacetate, pyruvate, and acetyl-CoA to sustain anabolic metabolism (Mullen et al., 2012). Although most cell culture-based studies of cancer metabolism have focused on the utilization and fate of glucose and glutamine, tumor cells *in vivo* have access to other sources of nutrients, like amino acids, to sustain the elevated proliferation rate. Measuring consumption and release profiles of metabolites from the NCI-60 panel of cell lines identified high correlation between glycine consumption and cancer cells proliferation rates (Jain et al., 2012). Exogenous serine uptake rate increases dramatically in tumor cells and deprivation of serine acts as a trigger to activate serine synthesis pathway and rapid inhibition of aerobic glycolysis, which results in an increased flux to the TCA cycle (Maddocks et al., 2012). Glycine and serine can be inter-converted by serine hydroxymethyltransferase (SHMT) and be used for one-carbon metabolism and nucleotide synthesis (Labuschagne et al., 2014; Boroughs and DeBerardinis, 2015). The directionality of this inter-conversion has critical effect on cancer cell proliferation. Exogenous serine can be used both for protein biosynthesis and it can be converted to glycine and one-carbon units needed for *de novo* nucleotide biosynthesis, whereas exogenous glycine cannot compensate for nucleotide synthesis (Labuschagne et al., 2014). These findings may reflect the fact that tumor cell proliferation is supported by serine rather than glycine. Glycolysis also plays an essential role for nucleotide biosynthesis (Lunt et al., 2015) and understanding relative consumption rate of exogenous amino acids compared to glucose-derived serine and glycine in transformed cells will be important.

In addition to glutamine, serine and glycine, other amino acids may also contribute to cancer cell proliferation. Branched-chain amino acids (BCAAs) are abundant amino acids in plasma (Stein and Moore, 1954; Meister, 1965), and growth of wild type IDH glioma, subgroup of brain tumors with poorest clinical treatment, is highly associated with expression of branched-chain amino acid transaminase 1 (BCAT1; Yan et al., 2009; Tönjes et al., 2013). Metabolomics profiling of patient-derived glioma samples also suggested correlation between increasing tumor grade and cysteine catabolism (Prabhu et al., 2014). Although, the current state of investigations suggest that amino acids primarily are used for protein synthesis in proliferating cells (Dolfi et al., 2013; Zhang et al., 2014), whereas catabolism of amino acids might be more important to generate ATP and maintain cellular redox state in nutrients limited condition.

In addition to the metabolism of carbohydrates and amino acids, lipids can also be used as an important fuel to supplement cancer cells proliferation requirements. Uptake of lipoproteins and free fatty acids (FFAs) from the bloodstream is the main source of satisfying lipid requirement in adult mammalian tissues. Although, fatty acids biosynthesis is limited to a subgroup of tissues, including adipose, liver and breast, reactivation of lipid synthesis is commonly observed in tumor cells with different sites of origin (Menendez and Lupu, 2007; Abramson, 2011). *In vitro*, glucose supplies significant amount of carbon needed for the de novo synthesis of lipids, however, in hypoxic condition or expression of oncogenic Ras, phospholipids uptake can contribute to lipid pools, compensating decreased flux of glycolytic carbon through pyruvate dehydrogenase (Kamphorst et al., 2013). Hypoxia may also change the fate of glutamine entering mitochondria by preferring reductive carboxylation to support tumor cell growth by activating fatty acid biosynthesis (Metallo et al., 2012; Mullen et al., 2012). Recently, regardless of relatively low levels in serum and culture media, acetate was proposed as an important carbon source for fatty acid biosynthesis and mitochondrial metabolism in hypoxic or highly glycolytic tumors. Acetyl-CoA synthetase enzyme (ACSS1; Björnson et al., 2015) and (ACSS2; Comerford et al., 2014; Schug et al., 2015) plays a key role in cancer cell proliferation, by capturing acetate and converting it to acetyl-CoA.

## Genome-scale modeling of cancer metabolism

Biological systems are complex interactive networks with interconnected set of components (e.g., metabolites, proteins, nucleic acids) and the function of the many different pathways connecting these components is highly regulated. Mutations may cause dysfunction of some of these regulatory or functional pathways, and this may lead to the emergence of dysfunctional phenotypes. Uncovering how these biological systems orchestrate their activities to support specific phenotypic transformation, e.g., normal to cancer, is a major challenge in medical science, and gaining insight into the mechanisms underlying these transformation may enable improved disease diagnostic, prognostic, and treatment strategies (Hyduke et al., 2013; Resendis-Antonio et al., 2015). Recent technological breakthroughs in high-throughput omics techniques and next generation sequencing (NGS) methods has transformed biomedicine into a data-rich discipline capable of providing deeper insights into phenotypic states of cells by simultaneous measurement of a large number of cellular components. However, analyzing very large sets of omics data with the objective to extract new biological knowledge is not a trivial effort. Despite this significant technological progress, we can still only measure fluxes in eukaryote cells for a limited number of reactions in central metabolism (Niklas et al., 2010). Systems biology approach in general, and genome scale metabolic models (GEMs) in particular, can facilitate this hurdle and bridge this gap by allowing multi-layer integration of omics data in the context of whole biological system (Mardinoglu and Nielsen, 2012; Mardinoglu et al., 2015; Yizhak et al., 2015). Furthermore, GEMs enable simulation of multi-species relationships in different metabolic states under dynamic environmental and genetic perturbations (Oberhardt et al., 2009; Shoaie et al., 2015; Zhang et al., 2015). In this context, GEMs are a powerful framework to analyze omics data in health and disease states and to investigate fundamental cellular mechanisms (Mardinoglu and Nielsen, 2015).

**Figure 2:**
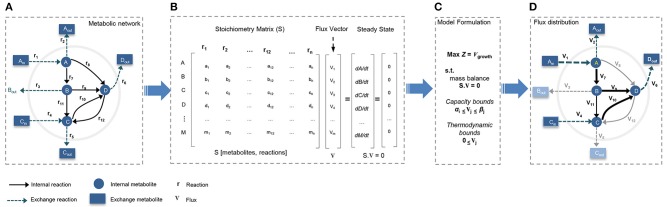
**Constraint-based modeling (CBM) and flux balance analysis (FBA)**. Genome-scale metabolic models have been constructed through constraint-based modeling approach and analyzed following FBA method to find feasible flux distribution. **(A)** Conceptual illustration of simple metabolic network by defining system boundaries, external/internal metabolites, exchange reactions, and internal reactions. **(B)** The stoichiometric matrix of the network is reconstructed to formulate FBA model under steady-state condition. **(C)** Models is formulated by defining a biologically/context relevant objective function and introducing physico-chemical constraints. **(D)** FBA provides an optimum feasible flux distribution relevant to defined objective function and compatible with enforced constraints.

Reconstruction of a GEM is performed by assembling biochemical transformations, occurring within a specific cell or tissue into the metabolic network (Thiele and Palsson, 2010; Mardinoglu and Nielsen, 2015). In GEMs the constructed stoichiometry matrix of the network covers stoichiometric coefficients of the metabolic reactions supplemented by detailed mapping of the protein coding genes to their corresponding reactions. In general, the metabolic networks are assumed to be modeled in quasi-steady state, and reconstructed GEMs are analyzed with constraint-based modeling (CBM) techniques (Figure 2). CBM shapes feasible solution space by imposing physico-chemical constraints, including thermodynamics, mass balance, and minimum/maximum flux capacity boundaries. The generated models usually remain under-determined, with possible alternative flux distributions satisfying the constraints. Flux balance analysis (FBA) method is used to formulate the problem and select an optimal flux distribution by optimizing for an objective function such as maximum biomass yield or ATP production (Thiele and Palsson, 2010; Mardinoglu et al., 2013b; Simeonidis and Price, 2015; Yizhak et al., 2015).

To date, a number of generic GEMs of human metabolism including Recon1 (Duarte et al., 2007), Recon2 (Thiele et al., 2013), HMR (Mardinoglu et al., 2013a), and HMR2 (Mardinoglu et al., 2014) have been reconstructed (Duarte et al., 2007; Thiele et al., 2013; Mardinoglu et al., 2013a, 2014). These models represent an assembly of all reactions documented to take place in metabolism of human cells/tissues integrated with known genes catalyzing each reaction, and have been used to generate context based GEMs of healthy human cells/tissues as well as transformed cells. In recent years, availability of cancer related high-throughput omics data made it possible to map this data into the generic human GEMs and to reconstruct cancer-specific genome-scale models. Several methods aiming to acquire tissue-specific or condition-specific active metabolic networks from a generic model have been developed. One of the first attempts in this context was the Gene Inactivity Moderated by Metabolism and Expression (GIMME) algorithm, which uses mRNA expression data as input together with presumed metabolic objectives to develop the context-specific reconstructed models (Becker and Palsson, 2008). Shlomi et al. (2008) proposed a computational method to generate tissue-specific metabolic networks by integrating tissue-specific gene and protein expression data with generic human genome-scale metabolic network. Same authors developed another method to reconstruct tissue-specific genome-scale metabolic models by integrating transcriptomics, proteomics, metabolomics, and phenotypic data. They use their method to reconstruct a functional metabolic model of human hepatocytes (Jerby et al., 2010).

Folger et al. (2011) has used a core set of 197 highly expressed enzymes common to at least 90% of the cells in NCI-60 cell lines database to generate a generic genome-scale metabolic model of cancer. This model has been used to capture core metabolic functions shared by cancer cell lines and to identify potential anti-cancer drug targets (Folger et al., 2011). Moving forward, Agren et al. (2012) reconstructed active metabolic networks in genome-scale for 69 healthy and 16 cancer cell types based on protein abundance data. A comparative analysis of generated models allowed prediction of cancer-associated metabolic features with potential to be used in identification of novel drug targets (Agren et al., 2012). Similarly, Wang et al. (2012) reconstructed GEMs for 26 human cancer and counterpart normal tissues, inferring tissue-specific metabolic models based on network topology and gene expression data. Pathway-level analysis of GEMs revealed eicosanoid metabolic pathway as a potential selective drug target (Wang et al., 2012). Jerby et al. developed a method to infer metabolic phenotypes by integrating transcriptomics and proteomics data derived from breast cancer patients into the GEMs. They identified a tradeoff linking decrease in tumor cells proliferation rate to evolved metastatic ability, as well as, to increased need for ROS detoxification (Jerby et al., 2012). Later, Goldstein et al. in an attempt to identify the role of p53 in regulating metabolic pathways, employed constraint-based modeling to characterize metabolic changes imposed by varying p53 status in human liver-derived tumor cells. Their results suggested that p53 may regulate glucose metabolism by preventing it to be shunted to growth promoting pathways such as glycolysis and the pentose phosphate pathway (PPP) and therefore inhibiting tumorigenesis (Goldstein et al., 2013). Feizi et al. used GEMs generated based on NCI-60 cell lines database to identify growth- associated metabolic sub-networks. They suggested critical role of concurrent synthesis and degradation of lipids in supplying energy for cell growth, and negative correlation of growth-associated sub-networks with colon cancer patients' survival (Feizi and Bordel, 2013). In addition to the cancer specific GEMs, Agren et al. (2014) reconstructed personalized cancer GEMs for 27 hepatocellular carcinoma (HCC) patients and used these models to predict 101 common and 46 patient-specific potential antimetabolites that could inhibit tumor growth in HCC patients. Recently, cell line-specific GEMs (CL-GEMs) were developed and successfully employed to identify cell-level metabolic phenotypes and selective drug targets (Yizhak et al., 2014; Ghaffari et al., 2015). We recently generated CL-GEMs for 11 human cancer cell lines with different site of origin and used them to investigated expressional heterogeneity of metabolic pathways across cancer cells. Furthermore, we used CL-GEMs to identify antimetabolites aiming to simultaneous inhibition of multiple growth supporting enzymes in proliferative cells. We predicted 60 common and 15 cell/cell group-specific potential antimetabolites and experimentally validated one of the identified anti-growth factors.

These studies demonstrate the successful employment of GEMs in deciphering metabolic foundations of neoplastic phenotypes and identification of new selective biomarkers and drug targets. Next generation of GEMs that integrate the cell metabolism with regulation and signaling are crucial but nontrivial challenge ahead, to tackle complexity of cancer cell metabolism. Up to now, a couple of methods have been developed to integrate the dynamic behavior of regulatory, signaling and metabolic networks in prokaryotes, and single-cell eukaryotes. The integrated FBA (iFBA) approach was used to generate an integrated model of central carbon metabolism in *Escherichia coli* by introducing ordinary differential equations (ODEs) and regulatory Boolean logic into FBA (Covert et al., 2008). Integrated dynamic FBA (idFBA) method proposed a FBA-based framework by incorporating metabolic, regulatory, and signaling processes through an integrated stoichiometric formalism, assuming fast reactions in quasi-steady state condition and introducing slow reactions in a time-delay manner (Lee et al., 2008). idFBA was applied to evaluate the phenotypic effects of environmental cues on *Saccharomyces cerevisiae*. However, large number of parameters required to be considered and accurate prediction of all the rate constants for the regulatory and signaling pathways is a long-standing challenge for this kind of approaches.

## Cancer metabolic heterogeneity, tumor microenvironment, and site-of-origin

The progressive accumulation of knowledge of cancer biology has demonstrated that cancer is an extraordinarily heterogeneous and complex disease. Tumors evolve through a reiterative process of clonal extension, genetic diversification and selection within the highly dynamic and adaptive ecosystem of target tissue (Greaves and Maley, 2012). Cancer cells rewire their metabolism to satisfy demands of cell proliferation and survival within the constantly changing tumor microenvironment (Hanahan and Weinberg, 2011). Despite common tendency of cancer cells toward uniformity of metabolism to support growth stimulating adaptations, variable patterns of clonal architecture, genetic diversity, and environmental effects results in a heterogeneous metabolic signature of tumors (Greaves and Maley, 2012; Meacham and Morrison, 2013). Apparently, a single model of cancer metabolism cannot describe the diversity of metabolic alterations happening during tumor progression. Differences in tumor micro-environmental conditions and also nutrients availability may constrain the potential metabolic repertoire of the cancer cells. *In vivo*, nutrient availability varies for different cell types, inside and across tissues/organs. For example, the structure of liver enforces gradients of nutrients and oxygen accessibility through hepatocyte zones (Puchowicz et al., 1999). Considerable expressional variation has been observed in cell cycle markers, together with association between oxygen gradients and hypoxia-related gene expression in glioblastoma cancer cells (Patel et al., 2014). Differences in active state of pyruvate kinase have been associated with proliferating and non-proliferating cell populations within breast cancer, revealing an influence of glucose metabolism on tumor formation and growth (Israelsen et al., 2013; Iqbal et al., 2014; Wong et al., 2015). Comparing expressional patterns of metabolic genes from 22 different human tumor types demonstrated overall similarity of gene expression program between tumors and corresponding normal tissue (Hu et al., 2013). Reconstructed CL-GEMs for 11 human cancer cell lines have been employed to investigate different mRNA expression pattern of metabolic pathways, and this revealed expressional heterogeneity of metabolic pathways across cancer types which can be exploited to identify generic or cancer-specific metabolic targets (Ghaffari et al., 2015). Site-of-origin may define the effect of oncogenic drivers in different tissues, as Myc-driven lung and liver tumors display dissimilar phenotypes related to glutamine metabolism (Yuneva et al., 2012).

Tumor-host metabolic interactions also have strong effect on the cancer cell metabolic reprogramming. Cancer cells may attempt to induce growth favoring conditions by actively manipulating the tumor microenvironment which can directly influence tumor progression, metastasis, and redox status (Guillaumond et al., 2013; Shukla et al., 2014). Metabolic cooperation between intra-tumor cell populations can help cancer cells to handle spatial heterogeneity of environmental conditions and nutrients availabilities. It has been shown that lactate out-flux by hypoxic pancreatic ductal adenocarcinoma cells can fuel the growth of neighboring normoxic cancer cells (Sonveaux et al., 2008; Guillaumond et al., 2013). Better understanding of tumor metabolic heterogeneity and tumor-host interactions has a high potential to optimize therapeutic strategies for selectively targeting cancer. Moreover, identification of the secreted proteins or peptides from cancer cells which remodel the tumor microenvironment may allow for discovery of novel cancer biomarkers and therapeutic targets (Feizi et al., 2015).

## Concluding remarks

Cancer cells are shown to experience characteristic changes in their metabolic programs, including increased uptake of glucose, enhanced rates of glutaminolysis and fatty acids synthesis, suggesting that metabolic shifts supports tumor cells growth and survival. Similarity some cancer-associated metabolic alterations are similar to the metabolic response of normal cells to growth-promoting signals, and this makes it difficult to separate neoplastic alterations clearly from the ones just reflecting increased cellular proliferation. However, different metabolic components target distinct oncogenic signaling pathways, and it is therefore important to elucidate the complex interaction between cellular metabolic network and oncogenic signaling network. Obviously, transformation from oxidative phosphorylation to aerobic glycolysis cannot simply explain the complete metabolic reprogramming event of tumor cells, and fairly little is known about different metabolic activities of cancer cells with diverse genetic and mutational background. Moreover, variances in metabolic profiles of tumors with same genetic lesion but different tissues of origin, suggests an open therapeutic window through investigation of complex metabolic interplay between tumor cells and stroma. It is becoming more evident that metabolic reprogramming in cancer cells is closely connected to hypoxia and ROS metabolism. Disrupting the balance between anti-oxidant production and increased biosynthetic activities in tumor cells seems to be a therapeutic opportunity.

Recent advances in high throughput omics technologies along with continuous progress in our understanding of cancer cell biology have portrayed a more complex and heterogeneous picture of tumor metabolism. Increased understanding of genetic and metabolic heterogeneity of cancer cells may open a new road toward development of new selective personalized diagnostic and therapeutic methods. GEMs, providing a mechanistic description of relationships between genes, metabolites and reactions within an interconnected functional metabolic network, have potential to integrate large-scale experimental datasets and extract knowledge out of their multi-dimensional complexity. Despite recent leap forward in employing GEMs to study cancer metabolism, more technical, and translational challenges are laying ahead. To date, the majority of models have been developed based on *in vitro* data that were produced under experimentally controlled conditions, but utilization of richer *in vivo* and clinical datasets, together with integration of cellular regulatory and signaling mechanism is critical for advancement of the field. Introduction of more metabolomics, fluxomics and growth related data along with other related omics data into the reconstruction and validation process of GEMs can help in increasing the accuracy and prediction power of these models. Furthermore, more and more data points toward the important role of tumor microenvironment in promoting plasticity and supporting cancer-associated metabolic adaptations, and modeling metabolic interaction between cancer and its environment can help in developing more effective therapeutic strategies. For this it may be necessary to integrate kinetics into stoichiometric genome-scale models.

In conclusion, metabolic reprogramming is crucial to support the uncontrolled proliferation, survival and migration of cancer cells, but at the same time renders tumors more vulnerable to metabolic perturbations. Identification of these metabolic dependencies at the genome-scale may provide an opportunity for optimized therapeutic intervention.

## Author contributions

PG, AM, and JN wrote the paper and all authors were involved in editing the paper.

### Conflict of interest statement

The authors declare that the research was conducted in the absence of any commercial or financial relationships that could be construed as a potential conflict of interest.
